# Biofilm Formation as a Complex Result of Virulence and Adaptive Responses of *Helicobacter pylori*

**DOI:** 10.3390/pathogens9121062

**Published:** 2020-12-18

**Authors:** Paweł Krzyżek, Rossella Grande, Paweł Migdał, Emil Paluch, Grażyna Gościniak

**Affiliations:** 1 Department of Microbiology, Faculty of Medicine, Wroclaw Medical University, 50-368 Wroclaw, Poland; emil.paluch@umed.wroc.pl (E.P.); grazyna.gosciniak@umed.wroc.pl (G.G.); 2 Department of Pharmacy, University “G. d’Annunzio” of Chieti-Pescara, Via dei Vestini, 31, 66100 Chieti, Italy; r.grande@unich.it; 3 Department of Environment, Hygiene and Animal Welfare, Wroclaw University of Environmental and Life Sciences, 51-630 Wroclaw, Poland; pawel.migdal@upwr.edu.pl

**Keywords:** *Helicobacter pylori*, biofilm, antibiotic resistance, outer membrane vesicles, coccoid forms, efflux pumps, biofilm matrix, quorum sensing

## Abstract

*Helicobacter pylori* is a bacterium that is capable of colonizing a host for many years, often for a lifetime. The survival in the gastric environment is enabled by the production of numerous virulence factors conditioning adhesion to the mucosa surface, acquisition of nutrients, and neutralization of the immune system activity. It is increasingly recognized, however, that the adaptive mechanisms of *H. pylori* in the stomach may also be linked to the ability of this pathogen to form biofilms. Initially, biofilms produced by *H. pylori* were strongly associated by scientists with water distribution systems and considered as a survival mechanism outside the host and a source of fecal-oral infections. In the course of the last 20 years, however, this trend has changed and now the most attention is focused on the biomedical aspect of this structure and its potential contribution to the therapeutic difficulties of *H. pylori*. Taking into account this fact, the aim of the current review is to discuss the phenomenon of *H. pylori* biofilm formation and present this mechanism as a resultant of the virulence and adaptive responses of *H. pylori*, including morphological transformation, membrane vesicles secretion, matrix production, efflux pump activity, and intermicrobial communication. These mechanisms will be considered in the context of transcriptomic and proteomic changes in *H. pylori* biofilms and their modulating effect on the development of this complex structure.

## 1. The Issue of Biofilm Formation in the Biomedical Sector

Despite their small sizes, microorganisms are characterized by exceptional complexity and diversity [1]. Classically, microorganisms can appear as free-swimming (planktonic) forms or multicellular clusters, referred to as biofilms [2,3,4,5,6]. Microbial biofilm is defined as a structured consortium of cells immersed in a self-produced matrix [2,6]. It is worth mentioning, however, that the biofilm may also include host components, e.g., fibrin, antibodies, platelets, or leukocytes. Biofilms can be attached to an abiotic or biotic surface, but they can also constitute a mobile, non-adhered structure floating in culture broth or body fluids [2,3]. Because of the proximity of microbial cells and their physical, metabolic, and social interactions, the biofilm growth differs significantly from the planktonic lifestyle [2,3,4,5].

Probably, the first observer of biofilms was Anthony van Leeuwenhoek (1632–1723), who, using a primitively constructed microscope, noticed microbial aggregates in oral samples [7]. The term “biofilm” was introduced by J. W. Costerton, in 1985, in a brief report presenting microscopic photos of *Pseudomonas aeruginosa* microcolonies residing in sputum samples of a cystic fibrosis patient. Although the presence of biofilms has its positive aspect in many ecosystems, these structures also exert their negative impact in the biomedical sector [4,8]. The ability of biofilm cells to survive in the environment with elevated concentrations of antimicrobial substances is termed “recalcitrance” and is a hallmark of treatment failures. In addition, biofilm cells have significantly lower sensitivity to unfavorable physicochemical factors and the activity of the immune system. The reduced susceptibility of biofilms results not only from the processes of passive tolerance to antimicrobials (decreased metabolism, matrix limiting the penetration of antibiotics, or presence of persister cells), but also from the processes of active increase in the number of resistant microorganisms (intensification of horizontal gene transfer (HGT) or induction of the hypermutable state) [4].

In association with the prevalence of biofilm formation and its negative impact on the infections treatment, in 2014, the European Society of Clinical Microbiology and Infectious Diseases (ESCMID) developed recommendations for microbiologists and physicians to improve the diagnosis of the most common infections accompanied by the biofilm presence [2]. The list of such diseases is wide and continues to grow, including: chronic otitis media/sinuses, endocarditis, lung infections associated with cystic fibrosis, urinary tract infections, vaginosis, osteomyelitis, chronic wound infections, catheter-related infections, and cancers of bile ducts and digestive system [4,5,8]. In the context of the last type of infections, attention should be focused strongly on spiral, Gram-negative rods colonizing the gastric mucosa—*Helicobacter pylori* [9,10].

*H. pylori* has the ability to reside in the stomach for many years, often for a lifetime [11]. Although the presence of this bacterium may be associated with a number of gastrointestinal diseases, including gastric ulcers and cancers [12,13,14], more and more often the protective role against developing extra-gastric diseases, such as allergy, asthma, inflammatory bowel disease, or multiple sclerosis, is indicated [15,16,17]. Survival of *H. pylori* in an unfavorable, gastric niche is enabled by an intensive urease secretion, a spiral shape, presence of numerous adhesins, and the production of cytotoxic proteins, e.g., vacuolating cytotoxin A (VacA) and cytotoxin-associated gene A (CagA) [18]. It is increasingly recognized, however, that the adaptive mechanisms of *H. pylori* in the stomach may also be linked to the ability of this pathogen to form biofilms [19]. Nevertheless, this theme is still rarely discussed by scientists and despite the availability of results from original articles the number of reviews and exhaustion of *H. pylori* biofilm topic are unsatisfactory.

Taking into account the above facts, the aim of the current review is to discuss the phenomenon of *H. pylori* biofilm formation and present this mechanism as a resultant of the virulence and adaptive responses of *H. pylori*, including morphological transformation, membrane vesicles secretion, matrix production, efflux pump activity, and intermicrobial communication. These mechanisms will be considered in the context of transcriptomic and proteomic changes in *H. pylori* biofilms and their modulating effect on the development of this complex structure.

## 2. Characteristics of *H. pylori* Biofilms Produced in Laboratory Conditions

The first study showing the ability of *H. pylori* to form biofilm took place in 1999 [20]. Since then, the knowledge on this subject has expanded significantly, and the presence of *H. pylori* biofilms has been demonstrated in numerous in vitro studies using plastic or glass surfaces [21,22,23,24,25], cell cultures [26,27], gastric biopsies from animals [28] or humans [29,30], and even vegetable surfaces [31] or water distribution tanks [32,33,34]. It is worth noting that initially *H. pylori* biofilms were strongly associated by scientists with water distribution systems and were considered as a survival mechanism outside the host and a source of fecal-oral infections [35]. In the course of the last 20 years, however, this trend has changed and now the most attention is focused on the biomedical aspect of this structure and its potential contribution to the therapeutic difficulties of *H. pylori* [22,23,36,37,38,39].

Under laboratory conditions, *H. pylori* biofilm may be formed during both liquid or solid cultures. In the first case, there are several types of biofilms that can develop under such conditions, i.e., a biofilm formed on the liquid-air surface (most often as a macroscopically visible ring, possibly accompanied by a pellicle development) [22,40,41,42], a biofilm formed as sediment of cells adhered to a solid surface (cells are completely immersed in the medium) [21,22,41], and a biofilm present as multicellular aggregates freely drifting in the liquid phase (cells are immersed in the medium but do not sink to the bottom) [22,41,43]. In the case of culture on a solid substratum, the so called “colony biofilm” [38,39] or “lawn biofilm” [44] are most often indicated. Since the vast majority of research on *H. pylori* biofilms focuses only on these formed during liquid culture, the data discussed in this review will mainly spotlight such types of bacterial communities.

Environmental and cultivation conditions constitute parameters that may affect the process of *H. pylori* biofilm formation in the laboratory [21,22,24,45,46]. Adhesion of *H. pylori* to the surface, a first stage conditioning the biofilm formation, largely depends on the flow rate (inversely associated with the adhesion) and the surface charge (the negative surface charge is preferred), while the ambient temperature does not significantly affect the adhesion [24,45]. The pH of the environment is a factor that modulates the subsequent processes of biofilm formation. It was found that with decreasing pH, the ability of *H. pylori* to produce this structure declined, especially at very low values [22]. Exposure of the preformed biofilm to a slightly acidified environment (pH = 5.5) had no effect on the *H. pylori* biofilm, but still, a highly acidified medium (pH = 3) eliminated this structure. These results indicate the existence of a regulator of *H. pylori* that senses changes in the pH of the local environment and modulates the ability of this bacterium to form biofilm, especially in its early stages. An example of this type of regulator is ArsRS, which conditions the maintenance of *H. pylori* in a planktonic form in the strongly acidic environment of gastric juice [41]. Its more detailed characteristics and influence on the formation of *H. pylori* biofilm can be found in later parts of this article (“Transcriptomic and proteomic analysis of *H. pylori* biofilm forms”). Apart from environmental conditions, the chemical composition of the culture medium and the incubation period are other important parameters influencing the biofilm of *H. pylori* [21,46]. Laboratory experiments showed that the low serum content (2%) and the three-day culture were optimal for the maximal growth of *H. pylori* biofilms [21]. Extending the cultivation time does not increase the size of the biofilm, and sometimes even reduces it [21,22,41,47], indicating the dynamics of biofilm production and the participation of dispersion in the late stages of biofilm maturation [48].

## 3. Transcriptomic and Proteomic Analysis of Biofilm *H. pylori* Forms

**Figure 1:**
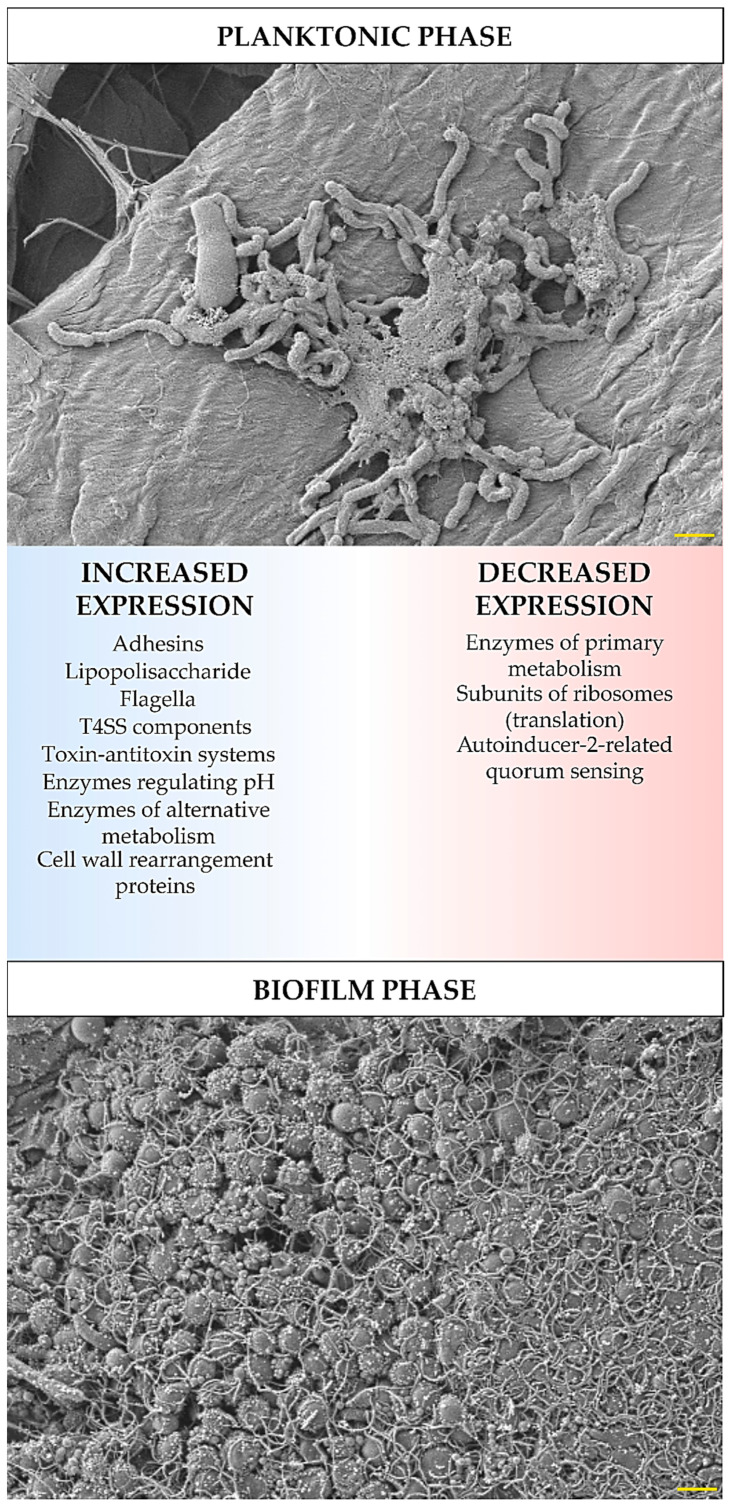
Diagram showing transcriptomic/proteomic changes in *H. pylori* cells during the transition from planktonic to biofilm phase. The scale bar shows 1 µm.

The development of biofilm structure and the transition from planktonic to biofilm phase are influenced by changes in the expression and translation of *H. pylori* genes. These changes were determined experimentally using ‘omics’ techniques (transcriptomics, proteomics, and metabolomics), and despite some differences between the results obtained by independent research teams, it is still possible to identify the most important features of the biofilm forms of this bacterium. In these studies, an increased expression has been demonstrated for: global pleiotropic regulators [21,22,49,50], adhesins (especially outer membrane proteins from the Hop and Hom family) [21,22,51,52], lipopolysaccharide (LPS) [21,22,51], efflux pumps [22,37,38,39,53,54,55], flagella proteins [21,43,51], components of T4SS systems [21,22,43,51], enzymes regulating pH (e.g., urease or arginase) [43,56] and responsible for obtaining alternative energy sources (e.g., hydrogenase) [22,43], proteins related to the cell wall rearrangement [22,43], and proteins of the toxin-antitoxin system [47] (Figure 1). Covering the components for which a decrease in the expression in biofilm *H. pylori* forms has been shown, these were factors involved in metabolism [21,22,57], translation [21,22], and quorum sensing related to the autoinducer-2 (AI-2) activity [22,46,48,55,58,59] (Figure 1). The influence of the transition of *H. pylori* into the biofilm form on the amount of antioxidant proteins is difficult to determine, because Yang et al. [56] and Shao et al. [43] showed their increase, while Hathroubi et al. [22] showed an opposite situation. It is possible that this difference results from the type of analyzed biofilm. Yang et al. [56] and Shao et al. [43] studied biofilm cells that were exposed to oxidative stress (air-liquid biofilms or these formed as microaggregates in the medium), while the one studied by Hathroubi et al. [22] was a sedentary biofilm produced at the bottom of wells, where the oxygen concentration is significantly lower.

Among the proteins mentioned above, it is worth paying attention to the production of global regulators of *H. pylori* physiology, because they will influence the expression of other genes under their control. In general, negative regulators bind to the promoter, reducing the attachment of RNA polymerase and lowering the expression of specific genes. In the case of positive regulators, binding is observed in the promoter’s upstream region, which determines the recruitment of polymerase and the increase in gene expression [60,61].

Shao et al. [43] showed that aconitase production increases in the biofilm phase. Aconitase acts as a pleiotropic regulator supervising antioxidant functions, motility and production of flagella, as well as the activity of some enzymes (urease and hydrogenase) [62]. It was established that the high activity of *H. pylori* hydrogenase is a feature of strains with a high carcinogenic potential [63]. Through hydrogenase, this bacterium can use molecular hydrogen (H_2_) to switch to the chemolithoautotrophic mode and use the energy from this process to bind CO_2_ [64]. Hydrogenase is also crucial for the functioning of the T4SS systems, both Cag-T4SS (involved in cytotoxicity and oncogenicity) and ComB-T4SS (involved in DNA transformation) [63]. It is worth noting that the ComB system is inactive at acidic pH and is stimulated in the environment with pH > 6.5, indicating that the transformation process takes place in close proximity to the gastric mucosa [65] and explaining the increase in urease production during biofilm formation (when microbial transformation is strongly intensified) [56].

The increased expression of *H. pylori* regulators during the transition to the biofilm phase, including HspR, HrcA, CrdR, and RsfS, was also demonstrated by the team of Hathroubi et al. [21,22]. HspR and HrcA have a positive effect on the production of flagella and selected adhesins, while negative on the chaperone proteins (GroES, GroEL, and DnaK) [66,67]. CrdR responds in *H. pylori* to the nitrosic stress (exposure to NO) [49]. It was experimentally found that flagellar components, iron transporter (*fecA*, HP0807) and efflux pump (*glnP*, HP1169) were among the genes with the highest induction controlled by CrdR [49]. For RsfS, participation in the inhibition of large and small subunits of ribosomes has been shown and, as a consequence, a reduction of protein synthesis (one of the most energy-consuming physiological processes) [68]. This mechanism seems to explain the observations of Hathroubi et al., who noticed a decrease in the expression of translation genes during the transition of *H. pylori* to the biofilm phase [21,22].

Apart from global regulators having a positive effect on the production of biofilm, it is also worth paying attention to the existence of the ArsRS regulator, which is a two-component system negatively associated with the production of this structure [41,52]. For ArsRS, the pH-dependent effect is indicated, during which exposure to the acidic pH of gastric juice inhibits the expression of genes related to biofilm formation, while in an environment with a neutral pH the effect of this system is inhibited [41]. The ∆*arsS* mutants showed a higher degree of adhesion to the surface and more intensive biofilm production (twice after 1–2 days and four times after 3 days of incubation than the wild-type strain) [41,52]. Moreover, in these mutants, an increase in the expression of genes encoding outer membrane proteins was noticed; these included *alpB* (omp21/*homB*), *sabA* (omp17/*hopP*), *labA* (omp2/*hopD*), and *hopZ* (omp1) [52,69].

The presented data show that global regulators can significantly influence the physiology of *H. pylori* and determine the transition from planktonic to biofilm form and vice versa.

## 4. Phenotypic Variability as a Modulator of *H. pylori* Biofilm Structure

**Figure 2:**
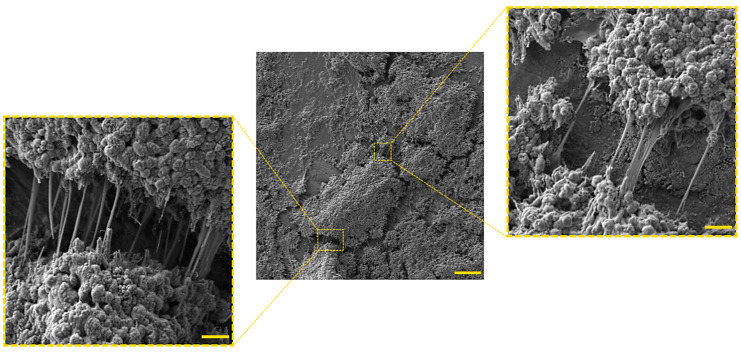
Representative photos showing a seven-day-old sediment *H. pylori* Tx30a biofilm along with the present exopolysaccharide matrix. The scale bar shows 40 µm for a central picture and 1 µm for magnified fragments of the photography.

**Figure 3:**
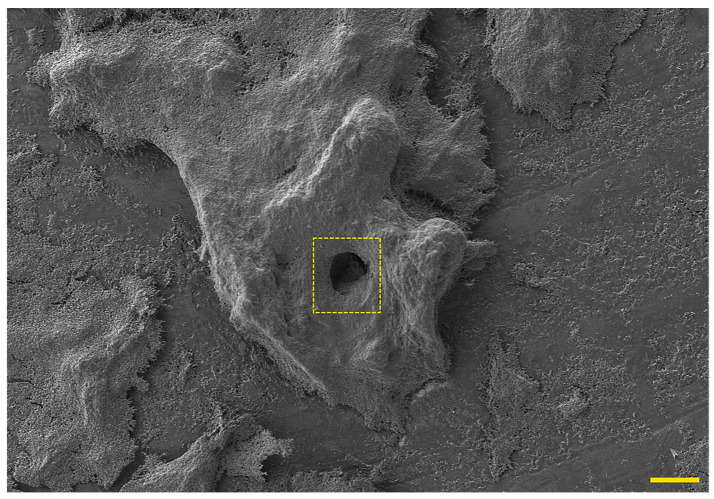
Representative photo of a mature seven-day-old *H. pylori* Tx30a biofilm formed on the liquid-air surface, in which the presence of a large water channel (marked with a dashed square) running through the biofilm structure can be seen. The scale bar shows 40 µm.

**Figure 4:**
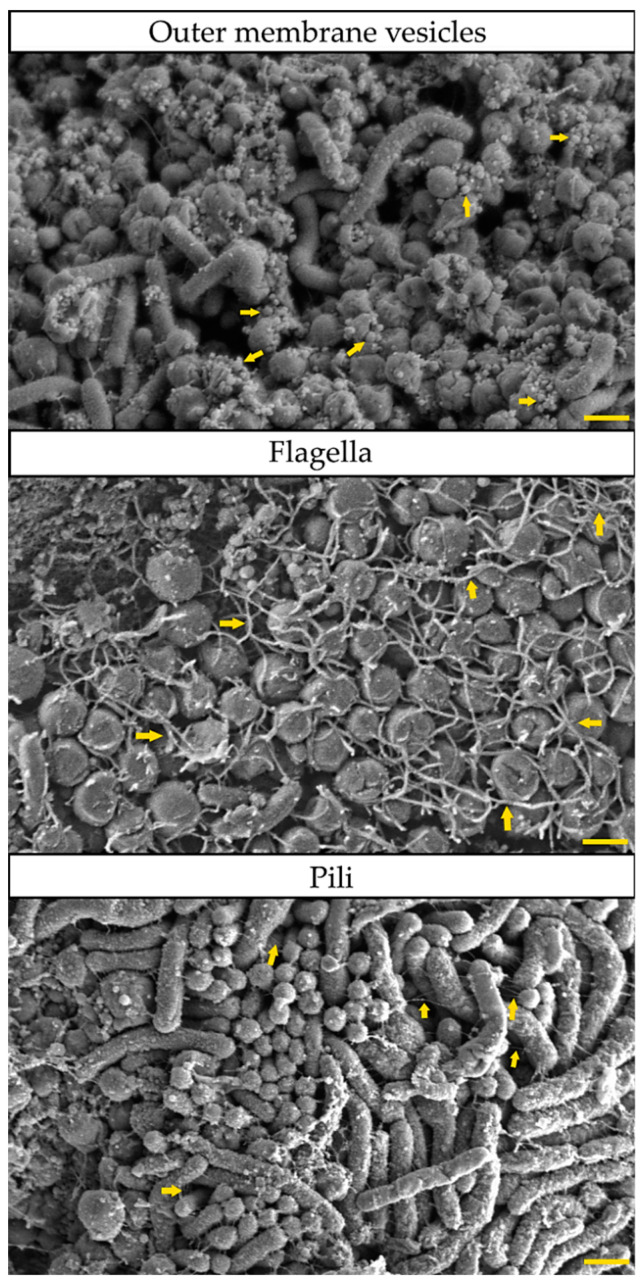
Representative photos of cellular and extracellular structures involved in the formation of *H. pylori* biofilm (based on multidrug resistant, clinical *H. pylori* 8064). From above: outer membrane vesicles (OMVs) visible as tiny, oval structures of 50–300 nm diameters; flagella noticeable as long structures entwining bacterial cells; and pili seen as very fine, thin filaments connecting bacteria cells. All the above-mentioned structures are marked with yellow arrows. The scale bar shows 1 µm.

Biofilm of *H. pylori* is a highly complex structure, consisting of morphologically heterogeneous bacterial cells immersed in the matrix (Figure 2), in which the presence of intercellular spaces and pores (water channels) is noticeable (Figure 3) [21,22,40,41,56,58]. In the *H. pylori* biofilm matrix, apart from bacteria, many cell structures can be found, including outer membrane vesicles (OMVs), flagella, and pili (most likely a component of *tfs4*-T4SS, a system associated with HGT [22]) (Figure 4). Both the morphotype of bacteria and the individual structural/extracellular components mentioned above can influence the process of biofilm formation and its architecture. Therefore, the function of each in the context of *H. pylori* transition to the biofilm phase will be described in the next sections of this review article.

### 4.1. Coccoid Forms

In the initial stages of biofilm formation, *H. pylori* is in a spiral form [45,56,58]. This morphological form is highly mobile and associated with the colonization of new niches [11,70]. After effective adhesion to the surface and multiplication, a morphological transformation occurs, which is accompanied by the creation of high shapes heterogeneity (spiral, rod-shaped, curved, coccoid, and filamentous forms) [70]. However, in the case of prolonged cultivation, all cells in the biofilm finally transform to a coccoid form, for which participation in survival and higher tolerance to adverse environmental factors is indicated [71,72]. It is worth mentioning that the morphological transition of *H. pylori* to coccoid forms is accompanied by numerous physiological changes, including reduction of cell size, limitation of intracellular ATP, changes in cell membrane potential, and generation of endogenous oxidative stress-dependent modifications in the structure of DNA and proteins [71]. These processes are associated with the acquisition of a viable but non-culturable (VBNC) phenotype by spherical *H. pylori* forms and loss of culturability [73], although there are also reports showing the possibility of the existence of spherical *H. pylori* forms in a culturable form [74].

A recently published report by Kadkhodaei et al. [74] has described an *H. pylori* strain with changed phenotype (overproduction of mucus on agar plates occurring only in a spherical morphotype; hence, it was defined as a mucoid-coccoid strain). In contrast to the parental strain with a typical phenotype and high antibiotic sensitivity, from which the *H. pylori* mucoid-coccoid strain was derived, the latter was resistant to all tested antibiotics. This shows the link between the overproduction of the extracellular matrix and the presence of spherical *H. pylori* forms with tolerance to antimicrobial substances.

Transcriptomic analysis by Poursin et al., determining the effect of the morphology change of *H. pylori* on the *spoT* status, showed 30 times higher expression of this gene in coccoid forms than in their spiral counterparts [75]. SpoT is a bifunctional enzyme responsible for both the synthesis and degradation of (p)ppGpp (referred to as alarmone). For SpoT, it has been shown that this enzyme contributes to the biofilm formation and supervision of efflux pumps, i.e., proteins associated with antimicrobial removal and resistance generation [39,76]. A detailed description of the involvement of efflux pumps in the formation of *H. pylori* biofilm and the induction of antibiotic tolerance will be presented in the “Efflux pump activity” section.

Apart from the increased level of SpoT, proteomic studies by Wuchta et al. [77] and Loke et al. [78] showed an increase in the production of the following components in spherical *H. pylori* forms: outer membrane proteins (Omp7 (HopF), Omp8 (HopG), Omp11 (HorE), Omp15 (HopE), and Omp25 (HopI)), transporter proteins/efflux pumps, flagellin A, urease and hydrogenase subunits, proteins regulating the metal ions concentration (e.g., NikR being crucial for urease and hydrogenase activity [79]), CagE and CagV (stabilizing the T4SS pilus biogenesis [80]), VacA (related to cytotoxicity and immunosuppression induction [81]), lipoprotein LPP20 and TNF-α-inducing protein (both contributing to the carcinogenesis promotion [82]), as well as MreB (influencing the action of urease, hydrogenase, and VacA [83]).

Research on the transcriptome/proteome of coccoid *H. pylori* forms and their influence on biofilm formation is a very difficult task. This is associated with the lack of a uniform procedure stimulating the transition of *H. pylori* from spiral to coccoid forms. Some reach teams use stressful conditions, such as nutritional starvation [43] or exposure to high oxygen concentrations [84], while others achieve this through a prolonged cultivation [77,78]. However, it cannot be denied that such procedures can independently affect the transcriptome and/or proteome of *H. pylori* cells. A review describing the difficulties in laboratory studies on coccoid *H. pylori* forms was published by Krzyżek and Grande (2020) [71]. Additionally, it is also worth mentioning that spherical *H. pylori* forms tend to self-aggregate and create biofilms [72,85], so it is difficult to distinguish planktonic and biofilm forms of this bacterium. According to the authors of this review, it seems that the processes of morphological transformation and biofilm formation of *H. pylori* are closely related and may induce one another, as observed in other microorganisms [86,87,88,89]. Spherical forms may stimulate biofilm formation through a high autoaggregation capacity, while biofilm may intensify the transition of *H. pylori* to spherical forms through the presence of niches with suboptimal growth conditions (e.g., reduced concentrations of nutrients or respiratory gases). Certainly, research in this area is absolutely necessary to better understand these processes.

### 4.2. Metabolic Activity and Matrix Production

Metabolomic studies on a selected collection of *H. pylori* strains, defined as weak and strong biofilm producers, showed that the former were characterized by a more intense and wider range of produced metabolites than strong biofilm producers [57]. These data are consistent with the observations of Hathroubi et al. [21,22], who showed a significant reduction in the expression of genes related to metabolism in biofilm *H. pylori* forms. Nevertheless, it should be remembered that biofilm forms are still metabolically active, and this activity may influence the composition of biofilm matrix [57].

Using confocal laser scanning microscopy (CLSM) and selective staining of biofilm components, it was found that the *H. pylori* biofilm matrix consists of proteins, extracellular DNA (eDNA), and sugars [21,24]. However, it was pointed out that proteins seem to be the most important units of the biofilm, because the treatment with proteinase K at any stage of biofilm development caused its significant degradation. The eDNA was also important (confirmed by the degradative activity of DNase), but only in the early stages of the biofilm formation.

In addition to the above illustrative results showing the components of *H. pylori* biofilm, others focused on the detailed chemical characteristics of this structure [20,56,90]. In the first study, it was determined that this polymer consists of various sugars, including fucose, glucose, galactose, N-acetylglucosamine, and N-acetylneuraminic acid, which largely constitute components of LPS and peptidoglycan [20]. Fatty acids were also represented by the compounds derived from *H. pylori* LPS, i.e., tetradecanoic acid (C_14_), hexadecanoic acid (C_16_), and octadecanoic acid (C_18_). Covering amino acids, these were predominated by: asparagine/aspartic acid, glutamine/glutamic acid, glycine, alanine, and leucine. Another study focused on the protein and sugar components of *H. pylori* biofilm [56]. The main sugar in mature biofilms was mannose (approximately 80%), the dominance of which was associated with numerous proteomannans (1,3- and 1,4-mannose bonds). Among proteins, attention was also paid to the importance of neutrophil-activating protein A (NapA) in biofilm formation (∆*napA* mutants showed reduced ability to create this structure) [56]. This may be linked with the strongly positive charge of this protein [91], explaining the preference of this bacterium for adhesion to negatively charged surfaces [24]. The last study determined surface-related *H. pylori* sugars, and found that these components mainly include LPS components, mannose-rich glycosides, and α-glucans [90]. A correlation was also found between the culture conditions (and thus the type of biofilm) and the sugar composition of the *H. pylori* cell surface. In strains grown on solid media, an increase in the number of amyloids (α-(1,4)-D-glucan amyloids) and a decrease in the number of LPS-related sugars was observed, while in liquid cultures the relationship was reversed.

All the above results indicate that the chemical components (their composition and quantity) may have a direct impact on the structure and stabilization of *H. pylori* biofilm. Moreover, many of these compounds are derived from structural organelles of bacterial cells. Thus, in order to better understand the complexity of the biofilm matrix, it is also necessary to take a closer look at the role and function of individual organelles in the physiology of *H. pylori*.

### 4.3. Secretion of Outer Membrane Vesicles

When considering the extracellular structures of *H. pylori*, which play an important role in biofilm formation, membrane vesicles should be mentioned. Membrane vesicles are spherical structures with nanometric dimensions that transport a variety of chemicals, delivering them to the target site in an effective concentration and conditioning protection against environmental factors, such as the action of nucleases and peptidases [92,93,94]. Membrane vesicles are produced in all phases of microbial growth and secreted by both Gram-positive and Gram-negative bacteria, including *H. pylori* [95,96].

**Figure 5:**
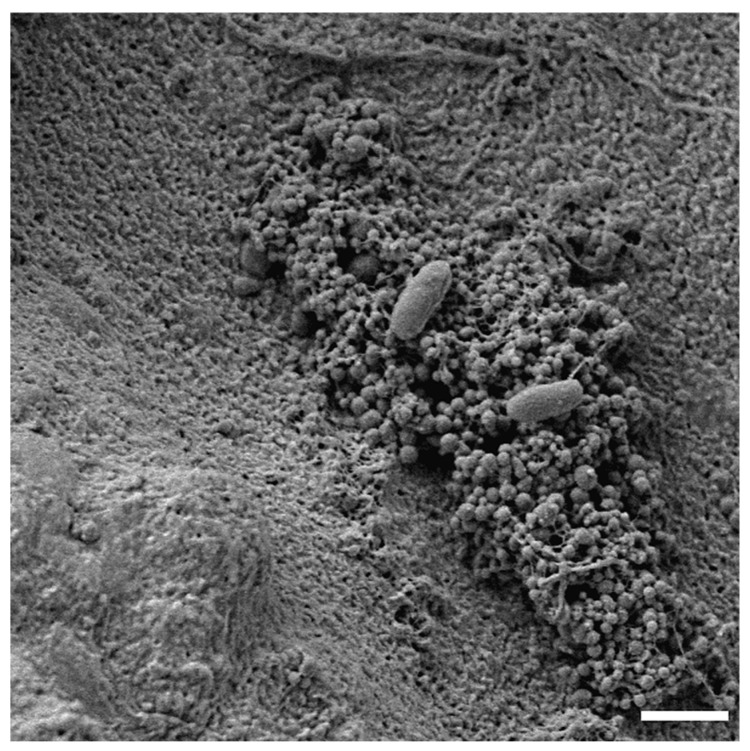
Representative photo of a clinical *H. pylori* 8064 strain selectively adhering to the surface of a high accumulation of outer membrane vesicles (OMVs). The scale bar shows 2 µm.

The research group of Grande et al. [97] found that significant amounts of eDNA can be found in *H. pylori* biofilm samples. However, the existence of denoting differences in intracellular and extracellular DNA profiles was determined with the help of random amplified polymorphic DNA (RAPD) analysis, indicating that cell lysis is not the main source of this component in biofilm. Further studies by this team determined that the vast majority of eDNA in *H. pylori* biofilm is related to OMVs [25]. It was also found that OMVs secreted by biofilm cells were secreted more intensively (approximately 24,800 OMVs/mL), had four times more eDNA, and possessed a more negative charge (−30 mV) than that produced by planktonic cells (18,000 OMVs/mL and −25 mV) [25,98]. On this basis, the participation of OMVs in genetic variation and scaffolding of biofilm has been suggested [25,97,98]. The assistance of these extracellular structures in the formation of *H. pylori* biofilm may play an important role in the early stages of this structure formation, which seems to be confirmed by the observations of Windham et al. [24] and Yonezawa et al. [99]. This could be related to the generation of negative surface charge by OMVs, which is preferred for *H. pylori* adhesion [24]. Although this type of mechanism has not yet been confirmed experimentally, the SEM analysis of *H. pylori* cells adhering preferentially to OMVs clusters seem to support the above hypothesis (Figure 5).

The involvement of OMVs in the formation of *H. pylori* biofilm was also independently investigated by a Japanese team led by Yonezawa et al. [99,100,101]. During the analysis of eight strains of *H. pylori*, it was noticed that one of them—*H. pylori* TK1402—was characterized by several times more intense biofilm production [99]. During microscopic observations, it was noticed that the main component of the matrix were OMVs. Further analysis of this group showed that OMVs from the *H. pylori* TK1402 strain possessed an outer membrane protein with a specific amino acid sequence [100,101]. This protein was Omp21 (also referred to as HopB or AlpB) and the variable region at positions 121–146 was crucial for its adhesive function [101]. In the comparison of *H. pylori* TK1402 with other strains, it was found that the former expressed *alpB* faster (24-h culture) than the others (48 h). Additionally, in *H. pylori* ∆*alpB* mutants a 10-fold reduction in biofilm formation capacity was demonstrated. The above data show that the proteins included in OMVs can play an important, eDNA-independent, structural function in *H. pylori* biofilm.

In proteomic studies of *H. pylori* OMVs, it was determined that the growth phase of this bacterium influences the size, composition, and preferential cargo of these extracellular structures [102,103]. Among 260 proteins found in OMVs, 171 were specific for these structures (not found in bacterial cells) [102]. Proteome of OMVs was represented by outer membrane proteins, efflux transporter/pump proteins, cytotoxic proteins (e.g., VacA and CagA), as well as numerous flagella components and enzymes with various activities, including catalase, urease, carbonic anhydrase, peptidoglycan modification enzymes, and beta-lactamases. It should be pointed out that many of these enzymes play an important role in the environmental adaptation of *H. pylori* and may be involved in the stress tolerance of this bacterium. The contribution of OMVs in the survival of *H. pylori* after exposure to oxidative stress and the action of cathelicidins or antibiotics has been initially confirmed in recent years [104,105], yet little is known about the molecular mechanisms governing these processes.

Regardless of this, the above studies show the importance of OMVs in the formation of *H. pylori* biofilm and the response to environmental stress through the assistance in the adhesion processes on the colonized surface and bacterial cells, participation in genetic recombination, and neutralization of harmful environmental factors.

### 4.4. Efflux Pump Activity

In the context of the protective function of *H. pylori* biofilm components, one cannot fail to mention efflux pumps and the influence of these structures on the antibiotic resistance of this pathogen. Efflux pumps are proteinaceous transporters anchored in the bacterial cell membrane, the amount of which is highly expressed, e.g., under exposure to antimicrobial substances [106]. Their activity is related to the removal of substances harmful to the microbial physiology into the extracellular environment, and therefore, they are currently considered as one of the most important factors determining the antibiotic resistance of microbes. Moreover, the efflux pumps can also participate in the export of factors related to the microbial communication (autoinducers) or substances constituting the building units of matrix, thus playing an important role in the process of biofilm development [107,108].

In many studies on *H. pylori* biofilms, it was found that cells in the biofilm phase are less susceptible to antibiotics [23,36,37,38,53,54,76]. It was determined that minimal biofilm eradication concentrations (MBECs) were several times higher than minimal bactericidal concentrations (MBCs) or minimal inhibitory concentrations (MICs) recorded for planktonic cells, although in some cases the values were dramatically different. In a study conducted in 2020, on a large pool of clinical *H. pylori* strains [23], MBECs for amoxicillin were shown to be 1000-fold higher than MICs for planktonic forms (16 µg/mL vs. 0.016 µg/mL). Additionally, MBEC values for clarithromycin and tetracycline were 31-fold higher, while for levofloxacin and metronidazole, they were increased by 16 and 8 times, receptively. On the basis of the obtained results, the correlation between MBECs and the biofilm production capacity of *H. pylori* strains (expressed as optical density of crystal violet stained-biofilms) was determined. There was a significant correlation between the biofilm amount and MBECs for amoxicillin, levofloxacin, and clarithromycin. However, no correlation was noticed when determining the MICs of planktonic forms, i.e., values that are classically checked in the laboratory diagnosis of *H. pylori* resistance. On this basis, the authors of the article postulated the need to verify MBECs in the routine *H. pylori* antibiotic resistance determination [23].

The efflux pumps play an important role in generating *H. pylori* biofilm resistance. Independent results of various research teams show that biofilm *H. pylori* cells possess a several-fold increase in the expression of genes encoding the efflux pumps compared to planktonic cells [37,38,39,53,54,55,76]. Such an observation was proven for 22 genes of the efflux pumps, including HP0471 (*kefB*) [38,76], HP0497 [38,76], HP0605 (*hefA*) [37,53,54,55], HP0939 (*yckJ*) [38,76], HP1165 (*tetA*) [39,54,55], HP1174 (*glnP*) [39,55], HP1327 (*crdB*/*hefG*) [37,53], HP1486 (*ybhS*) [39,55], and HP1489 [37,53]. In addition, it was found that the expression level of the efflux pump genes was higher in clinical, multidrug-resistant *H. pylori* strains than in susceptible isolates of this bacterium [38,39,76], showing a direct correlation between the production of transporter proteins and the resistance phenotype. It has also been shown that *spoT* is expressed at a higher level in coccoid *H. pylori* forms [75], and this gene plays an important role in controlling the biofilm structure and efflux pump activity [38,39,76]. Thus, in line with previous reports pointing to the important role of SpoT in the transition to the stationary phase of microbes [109], it is postulated that this protein is involved in the global monitoring of adaptability and response to environmental stress of *H. pylori*, including morphological variability, efflux pumps activity, and biofilm production.

### 4.5. Quorum Sensing and a Role of Flagella in Biofilm Formation

When describing the genes coordinating the transition of microorganisms from the logarithmic growth phase to the stationary phase, one should also mention *luxS*, a gene involved in the production of AI-2 and microbial communication (the so-called quorum sensing) [110,111].

For *H. pylori*, it has been shown that the expression of *luxS* increases in the initial stages of growth, reaches its maximum during the late logarithmic phase (around the second day), and then gradually decreases, presenting a minimal level of expression during the stationary phase [46,58,59,112]. This was also confirmed by the transcriptomic analysis of biofilm *H. pylori* forms showing a reduction in the expression of *luxS* and other genes related to communication with AI-2, i.e., HPG27_227 (*aibA*; a periplasmic uptake protein AI-2) and HPG27_526 (an exporter of AI-2) [22]. The potential inverse relationship between LuxS production and the transition to the biofilm phase was demonstrated with the use of ∆*luxS* mutants, which revealed several times higher biofilm production compared to the wild strain [26,40,48]. The observations of increased biofilm formation in *luxS*-nonproducing strains were associated with a reduction in the amount of Al-2, which is a stimulus to chemorepulsion (negative chemotaxis), and thus, an enhanced sedentary *H. pylori* lifestyle [26,48]. These suggestions were indirectly confirmed later when the decreased expression of genes encoding flagella components (including *flaA*, *flgE*, *fliI*, *motA*, and *motB*) was noticed in ∆*luxS* mutants [26].

New light was shed on the significance of flagella by Hathroubi et al. [21,22], who showed, during the transcriptomic analysis of planktonic and biofilm *H. pylori* forms, higher flagella gene expression in the latter group (even seven times higher for some genes). The obtained results were confirmed by this team during the observation of biofilm production in the ∆*fliM* (lack of flagella) and ∆*motB* (flagella present, but lack of motility) mutants, for which a 25-fold and 6-fold reduction in the amount of biofilm were shown, respectively [21]. On this basis, it was concluded that flagella is an important structural component of *H. pylori* biofilms [21,22].

The above results show the multitude of interactions taking place during the formation of the microbial biofilm and the difficulties in unambiguously assessing the usefulness or non-usefulness of *H. pylori* genes during the formation of this complex structure.

## 5. Holistic Model Describing the Transition of *H. pylori* into the Biofilm Phase

The process of *H. pylori* adhesion to the surface is stimulated by the negative charge of the substrate [24]; hence, the secretion of OMVs with an anchored eDNA (a molecule generating their negative charge) may facilitate the first stages of adhesion [25,97,98]. However, it should be remembered that this mechanism is not obligatory, but rather may help to facilitate adhesion. The interaction of OMVs with bacterial cells is conditioned by both the interaction of eDNA with many positively charged *H. pylori* proteins [113] and also directly by the presence of numerous adhesins incorporated into OMVs [101,102]. The processes described above take place in the early stages of biofilm formation (the microcolony formation), which seems to be confirmed by studies suggesting the importance of eDNA in the early but not late stages of *H. pylori* biofilm formation [24].

Further steps in biofilm development appear to be largely controlled by global regulatory proteins [21,22,39,43,52,112]. Within them, some are induced during the transition into biofilm phase, including HspR, HrcA, and CrdR, all of which increase the expression of adhesins and flagella [49,66,67]; aconitase stimulates the production of flagella and the activity of urease and hydrogenase [62]; RsfS inhibits metabolic intensity and proteins translation [68]; SpoT supervises the biofilm matrix production and the efflux pumps activity [39,76]. Other global regulators, such as ArsS and LuxS, both controlling the mobility and maintaining bacteria in planktonic forms, are inhibited [26,48,52,69].

During the maturation of the microcolony into biofilm, a high number of morphological transformations occur, which ultimately result in the presence of a large population of coccoid forms of this bacterium [70,71]. This morphotype is associated with an increase in the expression of adhesins, urease, and hydrogenase as well as transporter proteins/efflux pumps [75,77,78]. In addition, spherical forms intensively secrete OMVs, which additionally stabilize the biofilm structure, but also participate in the process of genetic recombination and tolerance to environmental stress (e.g., by the presence of numerous degradative enzymes) [25,104,105]. Efflux pumps, intensively expressed by the coccoid forms of *H. pylori*, take part in matrix production in the process of active ejection of building components into the extracellular environment and protect microbial cells against the bactericidal effect of antimicrobials [38,39,76]. Flagella, which are also highly expressed during biofilm formation, may be an independent, additional stabilizer of this structure [21,22]. Numerous protein components of *H. pylori* biofilms, including adhesins, flagella, or efflux pumps, are important at all stages of biofilm formation [21,22,24,36]. This is confirmed by the results showing the ability of proteinases to significantly degrade the biofilm of this bacterium both in the early and late stages of this structure development [24]. Sugar components are also part of the biofilm matrix and are mainly derived from LPS and peptidoglycan, indicating that the active OMVs secretion, efflux pump activity, and/or cell lysis contribute to the deposition of sugars in this polymer [20,90].

**Figure 6:**
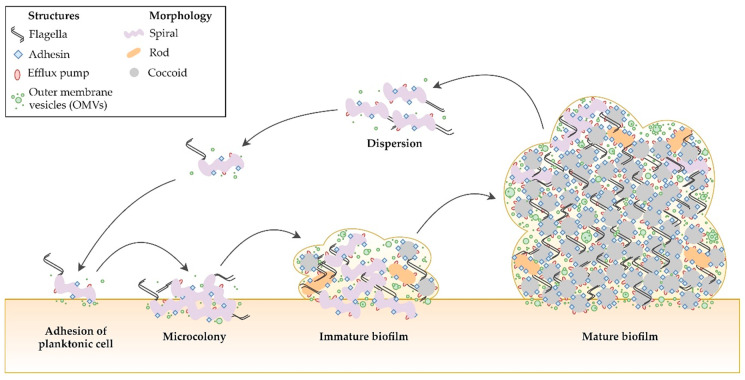
Stages of *H. pylori* biofilm development. Transition of bacteria from planktonic to biofilm phase is associated with the transformation of spiral forms into coccoids, as well as increased expression of structural components (adhesins, flagella, and efflux pumps) and intensified secretion of outer membrane vesicles (OMVs). All of them determine the stabilization of the *H. pylori* biofilm structure.

In summary, biofilm formation is a stepwise and highly complex process (Figure 6). A number of changes in the expression and translation of *H. pylori* genes appear to be involved in the transition from planktonic to biofilm phase. Moreover, the process of biofilm structure development is influenced by various environmental factors and these of microbial origin, i.e., virulence and adaptive changes (morphological variability, OMVs secretion, metabolism, and efflux pumps activity).

## 6. Limitations and Challenges in *H. pylori* Biofilm Research

There is no doubt that research of *H. pylori* biofilm is still in its infancy. Nevertheless, this subject, especially in recent years, has been gaining momentum. Therefore, it is worth paying attention to factors that may inhibit or slow down the speed of this development.

Unquestionably, transcriptomic and proteomic studies have great cognitive value in the analysis of *H. pylori* transition to the biofilm phase, which has been strongly outlined in this review article. It should be emphasized here that their results largely depend on the knowledge about the function of specific genes and proteins. As can be seen quite easily, even in recent ‘omics’ research on *H. pylori*, the function of about 1/3rd of the genes or proteins that are defined as being significantly altered in expression is unknown (termed as “hypothetical protein”) [21,22,77,78,102]. It seems, therefore, that the substantial amount of future research on *H. pylori* should focus on determining their role in the physiology of this pathogen.

Differences in the medium composition and culture conditions significantly affect the obtained results. This is particularly important in the case of ‘omics’ techniques, which, because of their high sensitivity, bring out all such differences. At this point, it is worth recalling the previously discussed example of discrepancies in the expression of genes encoding antioxidant proteins [22,43,56], which were most likely the result of the analysis of distinct types of *H. pylori* biofilms. Some groups studied biofilm cells formed as microcolonies or a pellicle at the air-liquid interface (both exposed to oxidative stress) [43,56], while others focused on biofilm created as a bacterial sediment (exposure to oxygen was minimal) [22]. In the opinion of the authors of this review article, the standardization of all research procedures is not necessarily needed, because their diversity is a factor resulting in various interesting observations. However, the research procedure and the type of the analyzed bacterial cells should be very precisely indicated each time, so that the subsequent analysis of the results by other research teams is as easy as possible.

Another future challenge is to establish a specific procedure to verify the sensitivity of *H. pylori* biofilms and incorporate it into the routine diagnosis of antibiotic resistance of this bacterium [23]. It is certainly a task that will neither be easy to perform nor achievable in the coming years. However, this challenge is worth to be taken, because more and more studies have indicated the protective role of *H. pylori* biofilm in antibiotic recalcitrance, especially in the course of therapeutically difficult, recurrent cases of infections caused by this microorganism. In contrast to the previous paragraph, here, the authors of the review strongly encourage the establishment of a single, well-defined procedure allowing the comparison of minimum inhibitory or bactericidal values of *H. pylori* biofilms between different research teams/diagnostic laboratories.

## 7. Conclusions

The process of transition of *H. pylori* from planktonic into biofilm phase is a stepwise and highly complex process. The most important features describing this process are:

1. Strong influence of virulence and adaptive responses (morphological transformation, membrane vesicles secretion, matrix production, efflux pump activity, and intermicrobial communication) on biofilm development and its structure.
2. Higher expression of adhesins, lipopolysaccharide, flagella, components of T4SS systems, toxin-antitoxin systems, efflux pumps, enzymes regulating pH (e.g., urease) and responsible for obtaining alternative energy sources (e.g., hydrogenase), and proteins related to the cell wall rearrangement.
3. Lower expression of factors involved in metabolism, translation, and quorum sensing related to the autoinducer-2 (AI-2) activity.
